# Optimization Strategies Used for Boosting Piezoelectric Response of Biosensor Based on Flexible Micro-ZnO Composites

**DOI:** 10.3390/bios12040245

**Published:** 2022-04-14

**Authors:** Xiaoting Zhang, Jose Villafuerte, Vincent Consonni, Eirini Sarigiannidou, Jean-Fabien Capsal, Alexis Bruhat, Daniel Grinberg, Lionel Petit, Pierre-Jean Cottinet, Minh-Quyen Le

**Affiliations:** 1 Electrical Department, LGEF, Université Lyon, INSA-Lyon, EA682, F-69621 Villeurbanne, France; xiaoting.zhang@insa-lyon.fr (X.Z.); jean-fabien.capsal@insa-lyon.fr (J.-F.C.); daniel.grinberg.pro@gmail.com (D.G.); lionel.petit@insa-lyon.fr (L.P.); pierre-jean.cottinet@insa-lyon.fr (P.-J.C.); 2 CNRS, Grenoble INP, LMGP, Université Grenoble Alpes, F-38000 Grenoble, France; jose.villafuerte@grenoble-inp.fr (J.V.); vincent.consonni@grenoble-inp.fr (V.C.); eirini.sarigiannidou@grenoble-inp.fr (E.S.); 3 CNRS, Grenoble INP, Institute NEEL, Université Grenoble Alpes, F-38000 Grenoble, France; 4 Department of Neurosurgery, Icahn School of Medicine at Mount Sinai, New York, NY 10029, USA; alexis.bruhat@mountsinai.org; 5 Sinai BioDesign, Icahn School of Medicine at Mount Sinai, New York, NY 10029, USA; 6 Department of Cardiac Surgery, “Louis Pradel” Cardiologic Hospital, F-69500 Bron, France

**Keywords:** flexible ZnO composites, micro-rod/particles, dielectrophoresis, piezoelectric biosensor, synthesis-based chemical bath deposition, characterization, simulation, medical application

## Abstract

Piezoelectric ZnO-based composites have been explored as a flexible and compact sensor for the implantable biomedical systems used in cardio surgery. In this work, a progressive development route was investigated to enhance the performance of piezoelectric composites incorporated with different shape, concentration and connectivity of ZnO fillers. ZnO microrods (MRs) have been successfully synthesized homogeneously in aqueous solution using a novel process-based on chemical bath deposition (CBD) method. The morphological analysis along with Raman scattering and cathodoluminescence spectroscopy of ZnO MRs confirm their high crystalline quality, their orientation along the polar *c*-axis and the presence of hydrogen-related defects acting as shallow donors in their center. The experimental characterizations highlight that ZnO MR-based composites, with a higher aspect ratio (AR), lead to a significant improvement in the mechanical, dielectric and piezoelectric properties as opposed to the ZnO microparticles (MP) counterparts. The dielectrophoretic (DEP) process is then subjected to both ZnO MP- and MR-based composites, whose performance is expected to be improved as compared to the randomly dispersed composites, thanks to the creation of chain-like structures along the electric field direction. Furthermore, a numerical simulation using COMSOL software is developed to evaluate the influence of the material structuration as well as the filler’s shape on the electric field distribution within different phases (filler, matrix and interface) of the composites. Finally, the aligned MR piezoelectric composites are revealed to be high potential in the development of innovative compact and biocompatible force-sensing devices. Such a technological breakthrough allows the achievement of a real-time precise characterization of mitral valve (MV) coaptation to assist surgeons during MV repair surgery.

## 1. Introduction

Zinc oxide (ZnO), given a non-centrosymmetric wurtzite structure, has been used extensively as a typical piezoelectric material in the fields of medicine, military affairs and telecommunication [1]. Compared to other piezoelectric ceramics or polymers (i.e., PZT, BaTiO_3_, PVDF) [2,3,4,5], it is desirable that no requirement of high-voltage poling for ZnO is needed to obtain piezoelectric capability. More importantly, the superior biosafety and biocompatibility of ZnO make it potential to engineer ZnO composites adopted in a biomedical environment [6,7]. Additionally, the ZnO nanostructures offer not only the miniaturization of many technologies but also size-enhanced properties with respect to bulk material due to the surface and confinement effects [8]. 

Recently, ZnO-based self-powered devices have been explored to harvest the biomechanical energy from in vitro environment without the need for external batteries (e.g., muscle stretching [9], body joint movement [10]) and from in vivo environment (e.g., breath and heartbeat [11]). In addition, ZnO-based devices would be an excellent implanted pressure sensor by detecting the dynamic mechanical deformation, e.g., the application of fraction flow reserve (FFR) technique in coronary stenoses [12]. Human health activities, such as sleep behavior, could be further monitored to aid diagnosis [13,14]. Hence, composite-based hybrid material, comprising ZnO micro- or nano-sized fillers incorporated into the polymer matrix, is clearly an interesting solution in the development of high-performance sensing devices. On one hand, the mechanical flexibility of the developed composites can be adjusted by choosing an adequate polymer matrix [15,16]. On the other hand, their dielectric and piezoelectric properties can be tailored by changing the size, shape, concentration and dispersion of fillers. 

In our previous studies, significant improvements in the dielectric and piezoelectric behaviors of ZnO composites have been confirmed by increasing the particle’s concentration [17,18]. Unfortunately, this improvement is limited due to the saturation of micro-particles (MP) concentration, in which obtaining a value beyond 44 vol.% is a real challenge. Alternatively, on the basis of the categorized theory developed by Newnham et al. [19] for identifying the arrangement of the constituent phase in multiphase composite systems, 1–3 connectivity composites where continuous fibers are vertically embedded from the bottom to the top layer in a polymer matrix can achieve attractive piezoelectric performance but are limited by their complicated fabrication and higher cost compared to the 0–3 composites [20,21]. An alternative approach relies on dielectrophoresis (DEP) for structuring material during the early curing stage, i.e., referring to the movement of polarizable particles induced by an alternating field [22]. The application of the DEP effect can be extended to assemble different sizes and shapes of particles, so as to form a chain-like structure inside a polymer matrix [23,24]. Indeed, the root cause of DEP assembly technology is the formation of a nonhomogeneous electric field owing to the dielectric contrast between the filler and polymer [25]. Thereby, tuning the external applied electric field involving the magnitude and frequency could change the DEP force driven on the particles. Those structured composites of ZnO MPs have been proved to exhibit enhanced dielectric, piezoelectric properties with regard to 0–3 composites with randomly dispersed particles [17]. Applications of material structuration are various [26,27], where 3D printing additive manufacturing could be feasible [28].

As opposed to a real 1–3 structure, quasi 1–3 composites created by DEP provoke interrupted filler phase within polymer interfaces [29]. The role of the interfaces between particles should be carefully considered, as it determines the portion of filler material along the electric field direction [30]. Therefore, the interfaces between particles with a larger number and longer distance can weaken the dielectric constant within each chain, resulting in decreasing the dielectric performance [31,32]. The analytical models developed by Bowen and Van den Ende [24,31] have demonstrated that the dielectric and piezoelectric constants of the structured composites are proportionately related to the ratio (γ) of the average particle size over the inter-particle distance along the aligned chains. Generally, the polymer interface is impacted by the filler geometry referring to the shape and size. For instance, composites of ZnO micro-sized particles have been characterized to possess higher dielectric constant than that of nano-sized particles owing to fewer polymer interfaces [17]. Alternatively, the inter-particle distance and the number of interconnections can be further decreased by aligning particles with a higher aspect ratio (AR) [31,33,34]. The AR is defined using characteristic dimensions of filler, i.e., equals to the ratio of the longest axis to the shortest axis. Meanwhile, during the DEP dynamic process, the fillers with a high AR orient with their longest axis parallel to the external electric field [35]. Thus, the application of ZnO microrods (MRs) whose AR is higher than that of spherical MPs would be a promising solution to improve the dielectric and piezoelectric performance of composites, but it has never been explored so far.

To further enhance the piezoelectric performance, our objective here involves the development of flexible composites consisting of ZnO MRs with higher AR than the former spherical MPs. Such new material design aims at creating fewer interconnections and a shorter inter-particle distance within the polymer matrix. DEP-processed composites with a quasi 1–3 structure are then characterized in order to confirm their performance. The effect of processing parameters (comprising amplitude and frequency of the applied electric field) on the alignment level is discussed and compared between composites integrating MPs and MRs. Numerical simulation together with empirical measurements allow a confirmation that the MR composites, with higher AR, give rise to a substantial performance enhancement with respect to the MP counterparts, particularly when being structured by DEP. Such an enhancement in the piezoelectric sensitivity, together with the biocompatibility of ZnO itself, could lead to the development of an innovative sensing device that allows surgeons to obtain precise physical and quantitative characterization of the coaptation (surface measurement and coaptation force mapping) throughout Mitral Valve repair (MVr) surgery. 

## 2. Fabrication and Methods of Characterization

### 2.1. ZnO MRs Synthesis 

**Figure 1:**
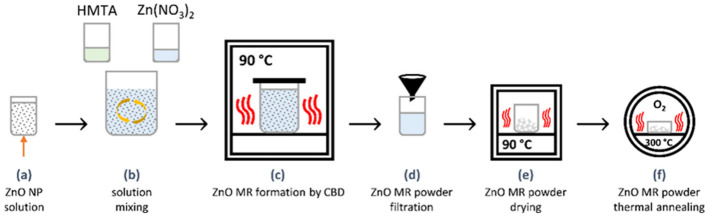
Fabrication steps of ZnO MRs: (**a**) Dispersion of ZnO NPs into deionized water via ultrasonication to form the ZnO NP solution; (**b**) Homogenization and mixing of the dispersed ZnO NP solution with the two precursor solutions to obtain the CBD solution; (**c**) Formation of ZnO MRs by CBD in a sealed reactor kept in an oven; (**d**) Filtration of ZnO MR powder using a nitrocellulose filter; (**e**) Drying of ZnO MR powder in an oven; (**f**) Thermal annealing of ZnO MR powder in a tubular furnace.

ZnO MRs were grown by chemical bath deposition (CBD) following and adapting a methodology reported in Refs. [36,37]. Commercially available suspended undoped ZnO nanoparticles (NPs) with a diameter lying in the range of 80–100 nm (Sigma-Aldrich) were used as nucleation seeds. The fabrication process of ZnO MRs is detailed in Figure 1. ZnO NPs with a mass of 240 mg were first dispersed in 100 mL of deionized water with the help of ultra-sonication for 30 min and second homogenized (Figure 1a). The ZnO NP solution was mixed with two separate precursor solutions composed of zinc nitrate hexahydrate (Zn(NO_3_)_2_·6H_2_O, Sigma-Aldrich, Burlington, MA, USA) and hexamethylenetetramine (HMTA, Sigma-Aldrich) in deionized water using an equimolar concentration of 30 mM (Figure 1b). The resulting CBD solution was stirred for 15 min and put in a sealed reactor. The sealed reactor was placed for 16 h in an oven kept at 90 °C (Figure 1c). The white powder composed of ZnO MRs was subsequently separated from the supernatant by vacuum filtration using a 0.1 µm pore membrane nitrocellulose filter (Whatman^®^, WHA7181004, MERCK, Darmstadt, Germany) (Figure 1d). washed with deionized water, and dried in an oven kept at 90 °C (Figure 1e). Thermal annealing was eventually performed for 3 h at 300 °C in a tubular furnace under oxygen atmosphere (Figure 1f). 

These above conditions for the thermal annealing were selected to improve the crystallinity of ZnO MRs and reduce the concentration of hydrogen-related defects acting as shallow donors. Villafuerte et al. pointed out that the tuning of the defects in terms of nature and concentration could be achieved using thermal annealing under oxygen atmosphere [38,39]. The study was carried out under various annealing temperatures ranging from 200 to 1000 °C. It was highlighted that the electrical properties of ZnO annealed at a moderate temperature of 300 °C specifically exhibit one of the smallest free charge carrier densities along with a high mobility, following the analysis of longitudinal optical phonon—plasmon coupling. Whatever the set temperature, the annealing process was performed for a short time of 1 h in order not to drastically change the sample’s morphology, especially under high temperature. In our case, as the temperature was moderate, a longer duration of 3 h was chosen to ensure perfect crystallinity and defect formation. 

### 2.2. ZnO MPs and MRs Based Composites 

To analyze the influence of the filler AR, two different shapes of ZnO microstructure were selected comprising MPs and MRs. Spherical ZnO MPs (~10 µm diameter, 99.9% purity) were provided by US Research Nanomaterials Inc. with a mass density of 5.6 g/cm^3^. These micro-sized powders were stored at room temperature and directly used to prepare the composite without any chemical modification. ZnO MRs were prepared by CBD as described above. The fabrication of the piezoelectric composites with ZnO MPs and MRs is based on the thin film casting method that has been reported in our previous work [17]. The matrix used to disperse the ZnO powders was polydimethylsiloxane (PDMS), i.e., commercial Sylgard-184 product purchased from Dow Chemical Company. The 0–3 ZnO MPs/PDMS and MRs/PDMS composite films with an 8% volume fraction were prepared and compared. Meanwhile, quasi 1–3 composite films integrating structured ZnO fillers were elaborated via DEP assembly technique. Thin-film sensors of 0.5 mm thickness were cut into circular samples of 16 mm diameter by using a home-made cylinder steel mold with a circular cavity of the same diameter. Thin gold electrodes of 12 mm diameter and 25 µm thickness were then deposited on two sides using a sputter coater (Cressington, 208HR, Ted Pella, Inc., Redding, CA, USA) under the condition of 0.4 mA and 60 s. Based on the MTM-20 High Resolution Thickness Controller, the 208HR system offers both uniformity and conformity of the coating electrodes, minimizing the charging effects. Moreover, as the thickness of the electrodes is neglected compared to the one of the film, they would not affect either dielectric or mechanical properties of the whole sample. Therefore, the characterization of the piezoelectric sensing performance is not influenced neither.

When exerting an alternating electric field on a ZnO filler suspension in an uncured thermosetting polymer matrix, randomly dispersed fillers coalesced to form chains, which were solidified within the matrix during the curing process. In order to investigate the effect of the input electric field on the alignment level, different amplitudes ($E_{app}=0.2,\;0.4,\;0.6\;V/µm$) and frequencies ($f_{app}=2,\;200,\;2000\;\mathrm{Hz}$) were applied on both DEP-processed ZnO MPs/PDMS and MRs/PDMS composites. $E_{app}$ was limited to 0.6 V/µm to avoid any electric breakdown of the samples, particularly in case of the MR composite, where a higher connectivity of fillers occurred with respect to the MP counterparts.

### 2.3. Characterization Methods

#### 2.3.1. Morphological Characterization 

The morphology and structural properties of ZnO MRs were investigated by field-emission scanning electron microscopy (FESEM) using a FEI Quanta 250 instrument and by transmission electron microscopy (TEM) using a JEOL-JEM 2010 microscope operating at 200 kV with a 0.19 nm point-to-point resolution. This equipment made it possible to achieve high-resolution microscopy (HRTEM) that allows for direct imaging of material morphology even on the atomic scale. The nature of nitrogen- and hydrogen-related defects was investigated by Raman and cathodoluminescence spectroscopy. Raman spectroscopy was performed using a Horiba/Jobin Yvon Labram spectrometer equipped with a liquid-nitrogen-cooled CCD detector. A 514.5 nm Ar^+^ laser with a power on the sample surface of ~0.51 mW was focused to a spot size of ~1 µm^2^ using a 100× objective lens. The integration time was 200 s per spectral window from 50 cm^−1^ to 3750 cm^−1^. A silicon reference sample was used for spectrum calibration at room temperature, with the theoretical Raman line set to 520.7 cm^−1^. The 5 K cathodoluminescence measurements of single ZnO MRs dispersed on SiO_2_/Si substrates were performed using a FEI Inspect F50 FESEM instrument equipped with a liquid-helium-cooled stage. The cathodoluminescence signal was collected through a parabolic mirror and analyzed with a 550 mm focal length monochromator equipped with 600 grooves/mm diffraction grating. Cathodoluminescence spectra were recorded with a thermoelectric cooled silicon CCD detector. A low acceleration voltage of 5 kV and a small spot size (i.e., less than 10 nm) were used to focus the acquisition on the single ZnO MR. The morphological properties of ZnO-based composites were studied by SEM using a Hitachi Flex SEM 1000 Ⅱ microscope.

#### 2.3.2. Mechanical, Dielectric and Piezoelectric Characterizations 

Mechanical compression tests were performed using a Shimadzu TCE-N300 instrument. Specimens were clamped between the compression plate kit and compressed at a speed of 5 μm/s under a displacement-controlled mode. Four compressive strain cycles (20, 40, 60, 70%) were exerted on the composites, and thus, the peak stress (occurred at the maximum strain) could be measured for a given strain.

Composites were clamped in a sample holder (AMETEK, Berwyn, PA, USA, 12962a) to measure their dielectric spectra with the help of a spectrum analyzer (Solartron, Bognor Regis, UK, 1255) and a dielectric interface (Solartron, 1296A). The measurements were performed using a driven alternating voltage (*V_RMS_* = 200 mV) in a large frequency range of 0.1 Hz to 1 MHz. 

The piezoelectric characterizations were performed in a specific test bench, which has been described previously [17,23,40]. In short, the samples were mechanically excited with a sinusoidal signal at 1 Hz and increasing amplitudes of force (F); meanwhile, the generated charges (Q) were synchronously collected and measured in a high-sensitivity charge meter (KISTLER, Type 5015) through a short-circuit loop. Thus, the charge coefficient *d*_33_ can be estimated as $d_{33}=\frac{Q}{A_{active}}*\frac{A}{F}$ where $A_{active}\;$ and A denote the surface of the gold electrode and the clamped surface, respectively. 

## 3. Results and Discussion

### 3.1. Morphological Properties of as-Grown ZnO MRs

**Figure 2:**
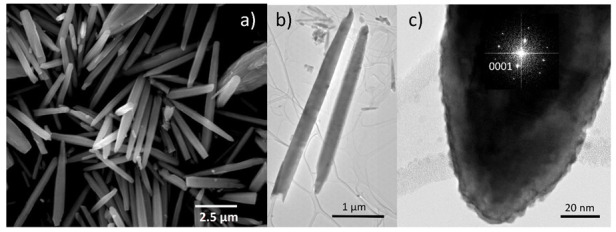
Morphological analyses: (**a**) FESEM image of dispersed ZnO MRs; (**b**) TEM image of two ZnO MRs; (**c**) HRTEM image of the ZnO MR grown along the polar *c*-axis and located on the right-hand side of (**b**). The corresponding FFT image is depicted as an inset.

**Figure 3:**
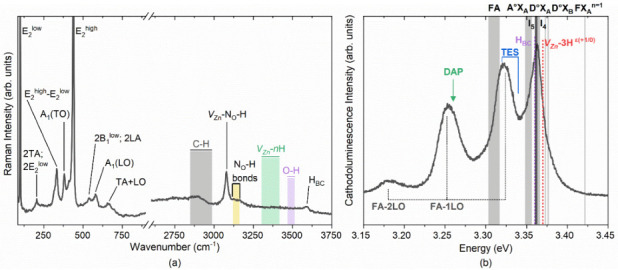
(**a**) Raman spectrum acquired on an array of undoped ZnO MRs grown by CBD and annealed for 3 h at 300 °C under oxygen atmosphere. The insets delimit the calculated and experimental vibrational frequencies from Raman and infrared absorption spectroscopy as deduced from Refs. [38,39,47,49,51,52,53]; (**b**) 5 K cathodoluminescence spectrum acquired on a single undoped ZnO MR grown by CBD and annealed for 3 h at 300 °C under oxygen atmosphere with a special emphasis on the NBE emission region. The insets represent the emission energy of optical transitions as deduced from DFT calculations and from experimental data in Refs. [38,39,54,55].

Figure 2a shows the FESEM image of the synthesized ZnO MRs grown by CBD under a magnification of 10,000. Through morphology analysis, it can be confirmed that the fillers have a rod shape, with a length of 3.9 ± 1.3 μm and a diameter of 477 ± 270 nm. The TEM and HRTEM images in Figure 2b,c reveal the elongated shape of ZnO MRs, which are grown along the polar [0001] direction (i.e., *c*-axis) as indicated by the inset representing the corresponding fast Fourier transform (FFT) image. The identification of intrinsic/extrinsic point defects incorporated in ZnO MRs grown by CBD is shown in Figure 3 by Raman and cathodoluminescence spectroscopy following the recent assignment of hydrogen- and nitrogen-related defects in ZnO grown by CBD, as reported in Refs [38,39].

The Raman scattering spectrum of annealed ZnO MRs is presented in Figure 3a and reveals their high crystalline quality through the highly defined phonon modes of the wurtzite structure in the low-wavenumber range of 50–900 cm^−1^. In particular, the E_2_^low^, A_1_(TO), E_2_^high^ and A_1_(LO) optical phonon modes are located at 99, 378, 438 and 574 cm^−1^, respectively. Second-order Raman lines, including 2TA/2E_2_^low^, E_2_^high^–E_2_^low^, 2B_1_^low^/2LA and TA + LO, are located at 203, 333, 536 and 666 cm^−1^, respectively [41]. No additional modes related to aluminum, gallium, antimony and/or iron dopants are detected [42], not even after thermal annealing, as expected by the CBD conditions for which a pH value smaller than 7 was used [43,44,45]. In the high-wavenumber range of 2750–3750 cm^−1^, several Raman lines associated with the presence of hydrogen, carbon and nitrogen impurities incorporated into ZnO MRs from the chemical precursors and medium are shown. The contribution of C–H*_x_* bonds (*x* = 1, 2, 3) is observed from 2750 to 3000 cm^−1^ [46]. The *V*_Zn_–N_O_–H defect complex is identified in the prominent Raman line at 3078 cm^−1^, along with the related N_O_-H lines at 3121 and 3160 cm^−1^ [39,47,48]. The interstitial hydrogen in the bond-centered site (H_BC_) defect is identified in the Raman line at 3575 cm^−1^ [49,50], while *V*_Zn_–*n*H defect complexes with *n* = 1, 2 and 3 give rise to several Raman lines from 3300 to 3418 cm^−1^ [49,51,52]. Interestingly, the relatively low intensity of H_BC_ lines correlated with the very low intensity of *V*_Zn_-*n*H lines follows the behavior of hydrogen- and nitrogen-related defects in ZnO grown by CBD and annealed for 3 h at 300 °C [39]. Given that H_BC_ and *V*_Zn_–3H defect complexes represent the major shallow donors responsible for the high density of free electrons in ZnO [3], it is deduced here that the thermal annealing of ZnO MRs favors a significant reduction in the density of free electrons, most likely below the typical value of 10^18^ cm^−3^ [39].

The cathodoluminescence spectrum of the annealed ZnO MRs, with a special emphasis on the near-band edge (NBE) emission region, is shown in Figure 3b. The NBE emission is dominated by radiative transitions involving neutral donor-bound A-excitons (D°X_A_) at ~3.363 eV. Specific contributions at 3.3628, 3.3614 and 3.360 eV are expected from H_O_ (I_4_), *V*_Zn_–3H defect complex (I_5_) and H_BC_ lines, respectively [38,54,55,56]. A prominent emission line is observed around 3.322 eV, which lies in the energy range of the two-electron satellite (TES) transitions from the I_4_ line [20]. 1LO and 2LO phonon replicas, separated by a phonon energy of 72 meV [20], are observed for this emission line. The broadening of these emissions could be expected from an important contribution of free-electron-to-acceptor (FA) [57] and donor–acceptor pair (DAP) [58,59,60] transitions related to nitrogen-doped ZnO. The appearance of phonon replicas may be related to a strong polar symmetry from A_1_(LO) and B_1_ phonon modes, as suggested in Ref [61] for this emission line.

It is worth noting that the significant Figure 3a,b of the two emissions experimentally measured were uniformed for a better analysis. The expected contributions quoted from the literature, however, cannot be uniformed, as the degree of precision varies.

### 3.2. Electric Field Distribution Based 2D COMSOL Model

In our previous work [17], we showed the influence of the electric field distribution on both 0–3 and 1–3 ZnO MPs composites during the DEP process. A similar study is reported here for the MR composites, with two different volume fractions of 2% and 8%. A higher volume fraction is difficult to obtain because of the agglomeration effect of MRs exhibiting a high surface energy. As a matter of fact, the MRs might precipitate out of the solution, resulting in a very high viscosity of dispersion that is unsuitable for composite fabrication. It is pointed out in this study that the MR composite, even integrating a low-volume fraction of ZnO fillers, could lead to similar piezoelectric performance as compared to the MP composite with a higher volume fraction of ZnO fillers. Actually, for a given ZnO concentration, the electric field applied on the MRs is much higher than that applied on the MPs. The following COMSOL numerical simulation confirms this observation.

**Figure 4:**
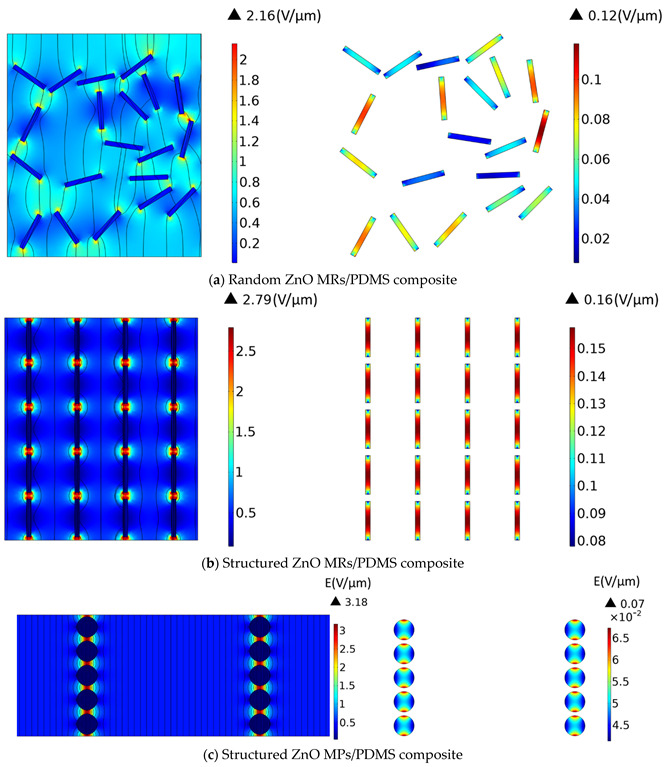
Electric field distribution in the three composites loaded with 8% volume fraction of ZnO fillers, all subjected to the same external excitation of 0.5 V/µm: (**a**) Random ZnO MRs/PDMS; (**b**) Structured MRs ZnO MRs/PDMS; and (**c**) Structured MPs ZnO MRs/PDMS. Two pictures are shown in each subfigure; the one on the left-hand side gives an overview of the whole composite, while the other on the right-hand side provides a closer view of the fillers. Both are not on the same color scale, for an easier visualization.

It has been revealed that the electrical properties related to the connectivity patterns of composites play an important role in their piezoelectric and dielectric properties [62,63,64]. To assess the electric field distribution within the fillers and their surrounding matrix, 2D finite-element models were built using COMSOL Multiphysics software. In order to highlight the effects of the material’s structuration, as well as the filler’s shape, three kinds of samples were drawn, as illustrated in Figure 4, i.e., comprising (a) random MRs composite, (b) structured MR composite and (c) structured MPs composite. All samples were elaborated with 8% volume fraction of ZnO fillers. The MR composites were assimilated to rectangle-shaped ZnO (3.9 μm length × 0.477 μm width) embedded in a PDMS polymer matrix (23 μm length × 20 μm width). To build the randomly dispersed filler distribution (Figure 4a), the filler’s orientation and position were pro-generated using MATLAB software with a random number generator function that can be then imported into COMSOL software. For the structured composites Figure 4b), rods were vertically aligned in one column and spaced equally at around 0.7 μm distance. This resulted in a ratio of the average particle size to the effective inter-particle distance (denoted γ) of approximately 0.18. The electric behavior of the composite was then evaluated via Electrostatics modulus, where the bottom was electrically grounded, and a floating potential was applied on the top to ensure an electric field of 0.5 V/μm across the composite. It should be noted that a periodic condition was applied on the sidewalls of the matrix to form a tangential continuous electric field along or across the side boundaries. Thus, this simple 2D geometry drawn in Figure 4 can be regarded as the repeating unit representing the whole composite material. The dielectric properties of ZnO and PDMS were initially extracted from the previous experiments [17,23]. 

The electric field distributions in random and structured MR composites with an 8% volume fraction are shown in Figure 4a,b, respectively. Owing to the polarizability contrast between the ZnO MRs and the polymer matrix, the electric field was greatly higher in the matrix than in the fillers, and the maximum values were focused on the interfaces between them. As expected, the electric field in either aligned fillers or interstitial matrix had a higher value than the one of the random sample. On one hand, the electric field in the structured composite converged into the narrower polymer gap in individual columns along the field direction, giving rise to the increased probability of electrical breakdown, especially under high input excitation. On the other hand, unlike the arbitrary electric field distribution in the random MRs (Figure 4a), the structured MRs exhibited a perfect symmetric distribution where all aligned fillers were subjected to the same electric field level (Figure 4b), In this case, ZnO experienced an insulating-to-conducting transition when the electric field exceeded the switching field (i.e., approximately 0.15–0.9 V/µm) [65,66]. Therefore, under the same input voltage (e.g., 0.5 V/µm), the structured MR composite led to an enhanced electrical conductivity, thanks to the formation of internal conduction paths driven from the filler’s alignment. Contrarily to the random sample, fillers were not well organized, and neither were the conduction paths, provoking inhomogeneous and inefficient electric field distribution. The electrical behavior undoubtedly affected the dielectric and piezoelectric characteristics of the composites, which were expected to be improved using DEP.

As a comparison between the MR and MP fillers, (Figure 4c), shows the electrical behavior of a structured particle composite, which is subjected to the same electric field input (0.5 V/μm) as in the case of the MR composite. The aligned ZnO circular-shaped MPs with a 10 µm diameter are embedded in a PDMS matrix with an identical volume fraction (8%) and a similar ratio γ (0.18). Although the polymer matrix of the MP composite obtains a slightly higher electric field than that of the MR composite (a factor of 1.1-fold), its value found on the MPs fillers is far lower as opposed to the MR fillers (a factor of 3-fold). This observation can be explained based on the distribution of the electric field profile within the rods, which is not similar to the case of particles. As demonstrated in (Figure 4b), on the right-hand side, the field is maximum at the center of the rods, slowly decreases on both sides and attains the minimum at the two extremities. Regarding the scale color, it can be affirmed that a high field level is concentrated on most of the MR surfaces. This is contrary to the MP, where only two maximum peaks are found at the two extremities, and a majority of the particles are stimulated by lower electric field levels. Accordingly, rod-shaped fillers seem to be more effective when combined with DEP. Such a high anisotropic-shaped filler is perfectly in accordance with the alignment direction, making it easier to successfully drive the electric field. Therefore, a higher strength of the electric field is obtained for the MRs, which somewhat decreases in intensity for the polymer matrix as compared to the case of the MP composite.

### 3.3. Optimization of DEP Processing Conditions 

**Figure 5:**
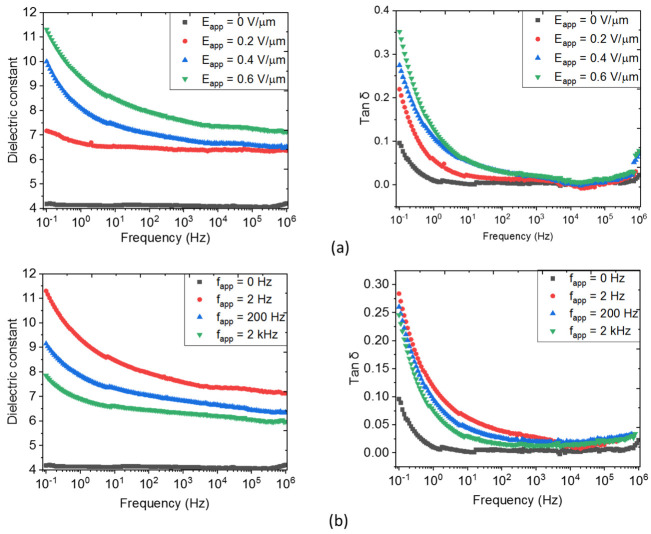
Broadband spectroscopy of an 8% vol. ZnO MP composite under different parameters of the applied electric field: (**a**) Varied amplitude and fixed frequency to 2 Hz; and (**b**) Varied frequency and fixed amplitude to 0.6 V/µm.

The degree of the filler’s alignment can be predicted through the measurement of the dielectric constants of the structured composite films [17,29,30]. Since the configuration of the DEP would affect the filler’s spatial distribution, it is necessary to assess the relationship between the dielectric properties of the composite and the parameters (i.e., amplitude and frequency) of the applied processing field. As observed in Figure 5, for both random and structured 8% vol. ZnO MP composites, the dielectric constant ($\varepsilon_{r}^{\prime }$) is almost unchanged under high frequencies (i.e., 1 kHz–1 MHz). In contrast, below 1 kHz and particularly under very low frequencies (i.e., 0.1–1 Hz), the variation in the dielectric spectra of the structured composite is considerable, which is ascribed to the contribution of high connectivity of ZnO fillers [17,18]. This behavior is confirmed by the significant change in the loss tangent (tanδ), i.e., originating from the conduction losses manifested essentially at low frequencies [67,68]. 

In Figure 5a, fixing the DEP frequency ($f_{app}=2\;\mathrm{Hz}$), the orientation angle increases with the electric field amplitude ($E_{app}$), which leads to higher $\varepsilon_{r}^{\prime }$ and tanδ. Contrarily, in Figure 5b, where $E_{app}$ is fixed at 0.6 V/μm), a decreasing trend of these dielectric parameters is observed, as the frequency $f_{app}$ is increased. Finally, the best processing parameters of the input electric field are found to be equal to 0.6 V/μm and 2 Hz, under which the dielectric constant of the structured sample achieves almost three times the enhancement as compared to the random sample (i.e., $\varepsilon_{r}^{\prime }=11.3$ versus 4.1 at 0.1 Hz). 

**Figure 6:**
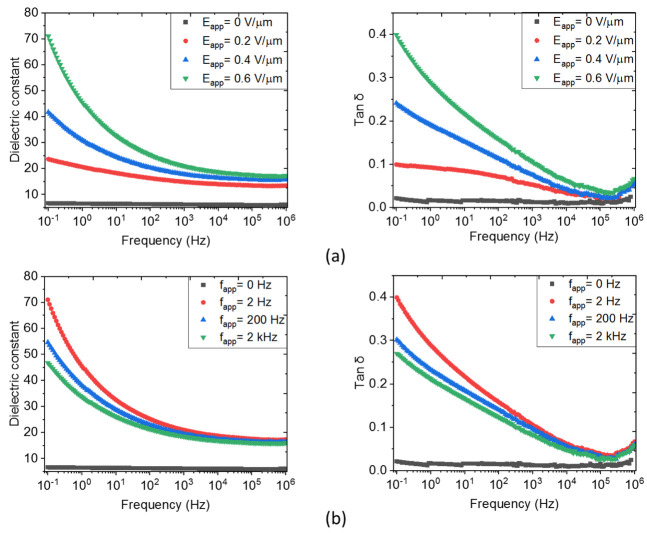
Broadband spectroscopy of an 8% vol. ZnO MRs composite under different parameters of the applied electric field: (**a**) Varied amplitude and fixed frequency of 2 Hz; and (**b**) Varied frequency and fixed amplitude of 0.6 V/µm.

To further boost the dielectric constant, MRs with a higher AR are incorporated into the PDMS polymer with an identical volume fraction (8% vol.), as shown in Figure 6, The ZnO MR composites are subjected to the same DEP configuration as in the case of the MP composites. Similarly, the dielectric constant of the MR samples would be enhanced by either increasing the amplitude or reducing the frequency of the electric field. However, for the MR composites, the frequency dependence of the dielectric parameters is not prominent as compared to the amplitude effect. For instance, changing the amplitude from 0.2 V/μm to 0.6 V/μm makes $\varepsilon_{r}^{\prime }$ of the aligned composite three-fold higher, increasing from 24 to 71 at 0.1 Hz. In contrast, a smaller variation in $\varepsilon_{r}^{\prime }$ (from 47 to 71 at 0.1 Hz) is recorded, as the frequency $f_{app}$ is decreased from 2 kHz down to 2 Hz. Interestingly, whatever the filler content, the random MR/PDMS composite still has higher $\varepsilon_{r}^{\prime }$ as opposed to the random MP/PDMS composite, confirming that the dielectric behavior is strongly impacted by the filler geometry [69]. Exceptionally, the MR composites structured by DEP substantially boost the dielectric permittivity, with a six-fold increase as opposed to the corresponding MR composite shown in Figure 5 (i.e., $\varepsilon_{r}^{\prime }=72$ versus 11.3). This increase is somehow more moderate in the case of the random samples (i.e., $\varepsilon_{r}^{\prime }=9.3$ versus 4.1). Therefore, it can be concluded that DEP is more efficient in structuring fillers with higher anisotropic shape. Regarding the tanδ trend, there is no obvious distinction between the MR and MP composites. Both lead to higher dielectric losses as the electric field amplitude is increased and remains unchanged with regard to the processing frequency. 

The results presented in Figure 5 and Figure 6 reveal that structuring material-based DEP leads to a substantial enhancement of the dielectric permittivity while simultaneously favoring the conduction losses at low frequencies. As higher conduction losses increase the probability of electrical breakdown during DEP, the optimization of the processing parameters must be considered to achieve the best compromise between the dielectric permittivity and tanδ losses. Consequently, an optimum tuning ($E_{app}$ = 0.6 V/μm, 2 Hz) is conducted on all particulate composites for further morphological and piezoelectric analyses, as reported in the following. 

### 3.4. ZnO MPs and MRs Based Composites 

**Figure 7:**
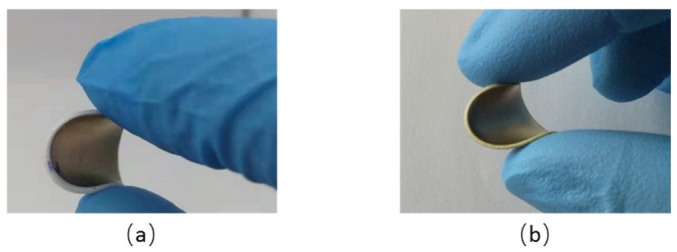
Elaborated flexible samples with 8% vol. of structured composite: (**a**) ZnO MRs/PDMS and (**b**) ZnO MPs/PDMS.

To better highlight the new insight of the paper, this subsection aims to demonstrate the influence of DEP-based structuration as well as the filler’s shape and concentration on the piezoelectric sensing performance of ZnO composites. The six samples used in experimental characterizations are: MPs/PDMS (two samples, structured/random with 8% vol. of ZnO) and MRs/PDMS (four samples of structured/random with 2% and 8% vol. of ZnO). Figure 7 represents two flexible ZnO composites incorporated with the same volume fraction of MR and MP fillers (8% vol.); both are structured through the DEP process. 

#### 3.4.1. DEP Assessment through SEM Image

**Figure 8:**
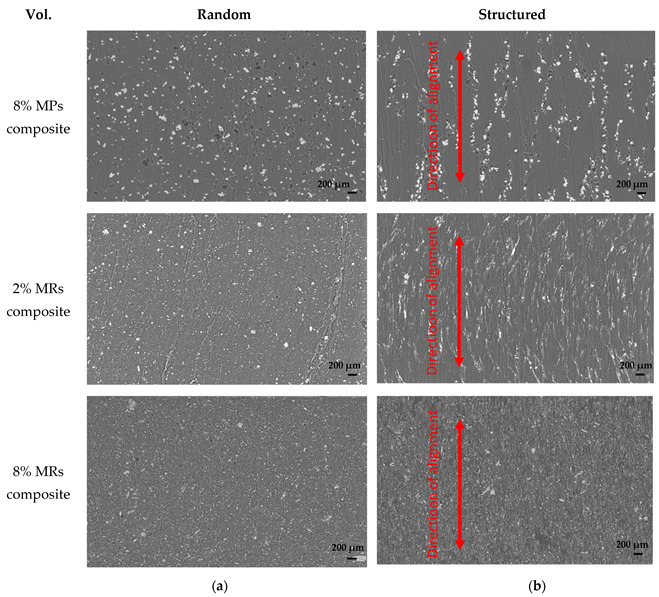
SEM images of (**a**) Random and (**b**) Structured composites integrating 8% vol. MPs, 2% vol. MRs and 8% vol. MRs under an applied electric field ($E_{app}=0.6\;V/\mu m$, $f_{app}=2\;\mathrm{Hz}$) during the DEP process (the scale bar is 200 µm).

The SEM results of the composites integrating different fillers are presented in Figure 8. Under the same input DEP conditions ($E_{app}=0.6\;V/\mu m$, $f_{app}=2\;\mathrm{Hz}$), the structured 8% vol. MP and 2% vol. MR composites clearly show the alignment of the fillers being shaped into a chain-like structure along the electric field direction. With a higher content of MRs (e.g., 8% vol.), however, the observation based on the SEM images is not obvious. Actually, the distance between the adjacent chains decreases as the filler’s concentration is increased, hence the inter-chain interactions are reinforced and overwhelm the dielectrophoretic force [70]. Accordingly, the morphology approach gives an overview of the material structuration under the DEP process and could clearly distinguish the difference in the filler’s shape (rod, particle). Nonetheless, this technique seems to be inadequate to accurately quantify the structuring effect of the composites, particularly with a high filler concentration. To achieve deeper and more convincing analyses, the measurements of the dielectric and piezoelectric characteristics of all samples are carried out in the following. 

#### 3.4.2. Mechanical Properties 

**Figure 9:**
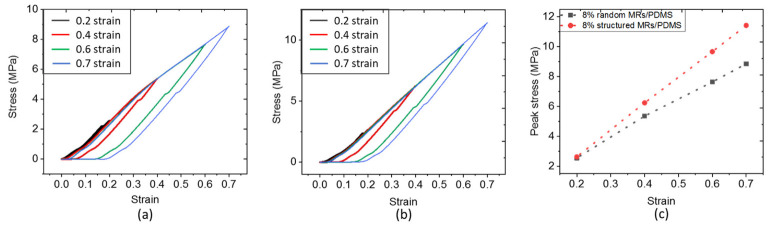
Compressive stress–strain curves of the 8% vol. (**a**) Random and (**b**) Structured ZnO MR/PDMS composites; (**c**) Peak stress versus strain for the 8% vol. random and structured ZnO MR/PDMS composites.

**Figure 10:**
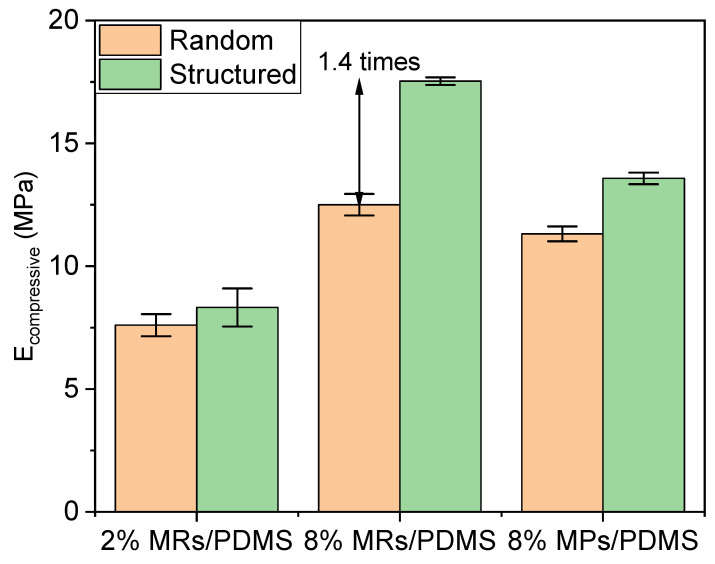
Compressive elastic modulus for different random and structured (i.e., aligned) ZnO MR/PDMS and MP/PDMS composites.

Firstly, the mechanical properties are discussed for the set of all samples due to their contributions to the piezoelectric behaviors. Figure 9a,b indicate the compressive stress–strain curves of the random and structured 8% vol. MR/PDMS composites, respectively. The composites experience a stepped loading profile, in which a series of fixed strains (including 0.2, 0.4, 0.6, 0.7) is exerted. After reaching the maximum value, unloading processes are conducted for a given deformation of the specimen. Both random and structured composites undergo a complete recovery, demonstrating how strong and flexible mechanical characteristics of the developed composites are. For a better evaluation of the compressibility, the peak magnitude of stress with the associated peak magnitude of strain are plotted in Figure 9c to derive the compressive elastic modulus (*E_compressive_*). As seen in Figure 10, *E_compressive_* of the structured (i.e., aligned) composites is enhanced and strengthened as compared to the random one, since the compressive load lies in the same direction as the applied electric field during the DEP process. In other words, the chain-like structure of the fillers makes the whole system stiffer in that direction. Among them, the alignment on the 8% vol. MRs-based composites during the DEP process can achieve 1.4-fold improvement as opposed to the random one. Additionally, under a volume fraction of 8%, composites of rod-shaped fillers are much stiffer than those of spherical-shaped fillers. Such an improvement is specifically evident for the Young modulus of the structured composites (~17.5 MPa), manifested by a better alignment of fillers, which in turn contributes to their excellent piezoelectric behavior.

#### 3.4.3. Dielectric Properties

**Figure 11:**
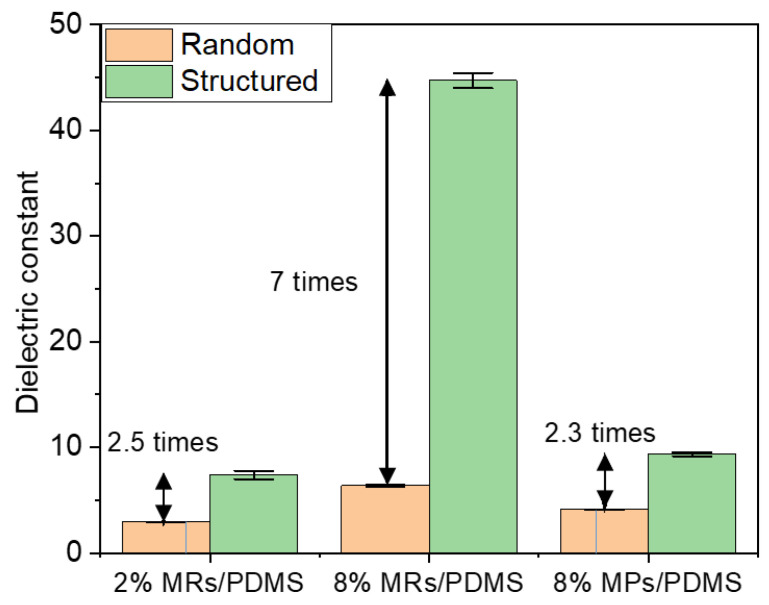
Dielectric constant measured at 1 Hz for different random and structured (i.e., aligned) ZnO MR/PDMS and MP/PDMS composites.

Secondly, the dielectric properties are discussed for the set of all samples. As seen in Figure 11, under the same optimal DEP conditions ($E_{app}=0.6\;V/\mu m$, $f_{app}=2\;\mathrm{Hz}$) and filler concentration (e.g., 8% vol.), the MP composites achieve around a two-fold increase in the dielectric constant ($\varepsilon_{r}^{\prime }$, measured at 1 Hz) with respect to the random one, while around a seven-fold increase is reached in the case of the MR composites. Hence, the improvement using the DEP process is more obvious in the composites fabricated with a higher AR filler. Interestingly, $\varepsilon_{r}^{\prime }$ of the low-fraction MR composites (2% vol.), either in the random or structured connectivity, is closed to the one of the high-fraction MP composite (8% vol.), suggesting an excellent efficiency of the MRs. Actually, considering the different dimensions in the MRs and MPs, the root cause of that improvement can be assigned to the shape (related to the AR) or the size effect. It has been demonstrated in our previous work that composites filled with nano-size particles possess a lower dielectric constant than that of micro-size particles, implying the suppressed effect of small-size filler [17]. Accordingly, it can be revealed that the dielectric property enhancement of composites integrating ZnO MRs is mainly impacted by their high AR, rather than their size. Indeed, during the DEP dynamic process, the high AR rods are found to be better aligned than the low AR particles. These observations are correlated with COMSOL simulations investigated in Section 2.3. Usually, spherical particles might be more easily attracted by their neighbors to form a corner-to-corner alignment, leading to an out-off-axis orientation effect [33]. Nonetheless, the high degree of alignment and fast orientation in the low AR particles seem to be easier to achieve compared to the high AR rods, which is due to the different hydrodynamic drag-induced torques [33]. This behavior should be considered in a transient regime, but after a long-time application of thermal heating and low-dynamic input field, a steady state is well established. The observation in that state clearly verifies that the composite embedded with high AR rods leads to higher dielectric performance. Alternatively, increasing the volume fraction of fillers, e.g., from 2% to 8% in our case, should be another effective way to improve the dielectric constant.

#### 3.4.4. Piezoelectric Properties

**Figure 12:**
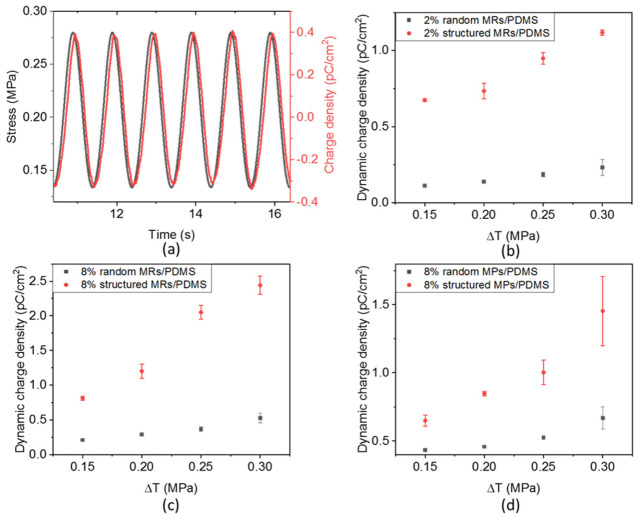
(**a**) Time evolution of the stress and generated charge density in the structured 8% vol. ZnO MR composites under a 0.15 MPa dynamic stress. Increasing dynamic change density (∆*D*) versus dynamic stress (∆*T*) in (**b**) 2% vol. ZnO MR/PDMS composites, (**c**) 8% vol. ZnO MR/PDMS composites and (**d**) 8% vol. ZnO MP/PDMS composites.

**Figure 13:**
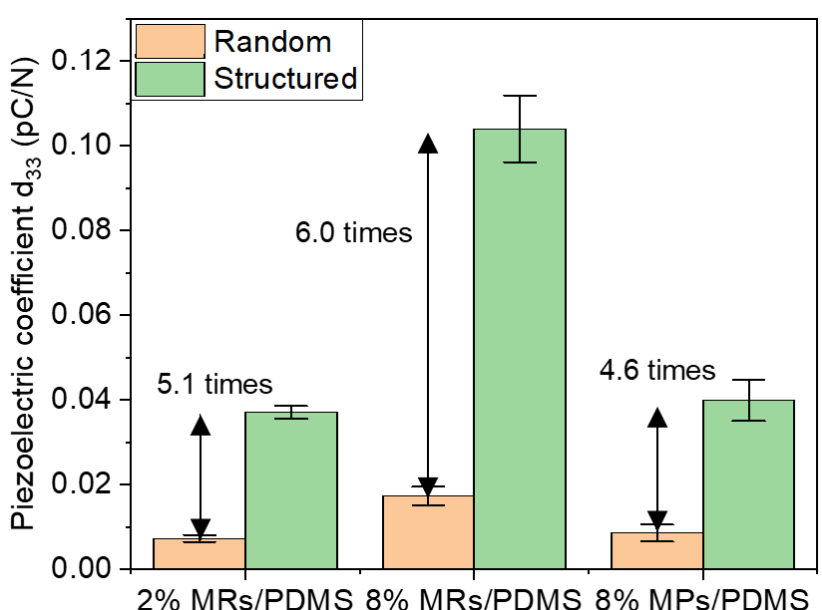
Piezoelectric coefficient (*d*_33_) measured at 1 Hz for different random and structured (i.e., aligned) ZnO MR/PDMS and MP/PDMS composites.

The piezoelectric behaviors of the composites were subsequently characterized by measuring the generated charge density (D) in response to an external oscillating stress (T) of 0.15 MPa magnitude and 1 Hz frequency. Figure 12a displays the time evolution of D and T for the structured 8% vol. MR/PDMS composites, reflecting the linear electromechanical coupling of a typical piezoelectric material. A small phase shift is found between D and T, which may be due to the detection delay or the restore/discharge mechanism of the capacitive sample. To better highlight the linear relationship between the electrical output and the mechanical input, Figure 12b–d illustrates the charge density variation (∆*D*) as a function of the dynamic stress variation (∆*T*) for the whole set of samples. The piezoelectric coefficient (*d*_33_) can be accurately deduced from the linear fitting of the Δ*D*-versus-Δ*T* characteristics, i.e., corresponding to the slope of the linear regression, as presented in Figure 13. These results confirm three possible approaches that can be used to improve the material performance:

- Enhancing the filler content gives rise to an enhanced piezoelectric response. For instance, increasing the volume fraction of the randomly dispersed MR composite (e.g., from 2% to 8%) leads to a 2.4-fold increase in the d_33_ value. However, it is challenging to fabricate high-density composites due to agglomeration and high viscosity effects.
- Integrating fillers with a higher AR could perform fewer interconnections between the neighboring phases, which in turn boost the piezoelectric effect. Using MRs instead of MPs allows the enhancement of the piezoelectric sensitivity of around 2.0-fold and 2.6-fold, respectively, for the random and structured composites integrating an 8% fraction vol. of ZnO filler.
- Structuring the filler dispersion via the DEP process leads to significantly enhanced piezoelectric properties. For example, under the same filler’s concentration, size and shape, the *d*_33_ value can be boosted even up to 6.0-fold for the 8% MR composites.

By comparing the above methods, the dielectrophoretic alignment seems to be the most efficient way to boost the dielectric and piezoelectric behaviors of ZnO composites. However, even with the optimized input parameters, it is difficult to achieve a perfect alignment of fillers. During the dynamic process of chain formation, the individual chains are attracted by adjacent chains to coalesce into a substantial columnar structure [71,72]. Additionally, the high connectivity from filler orientation in turn causes the conduction loss mechanism. Therefore, in the future, a realistic 1–3 composite structure, where vertically aligned ZnO NWs are embedded in the polymer matrix, appears as a promising route to further obtain a higher piezoelectric efficiency. 

## 4. Potential Application of Piezoelectric Biosensor on Force Measurement of Mitral Valve Coaptation

The materials reported in this research might have promising potential applications, involving in the development of biosensor device for cardiac surgery of mitral regurgitation (MRe). It has been well known that MRe is a major public health problem with a global prevalence of 9.3% in individuals over 75 years of age [73]. It is defined as reversed blood flow of part of the left ventricle load toward the left atrium at every cardiac systole, resulting in an overload in the left atrium and pulmonary circulation. MRe provokes symptoms such as shortness of breath and fatigue, provoking progressive heart failure and death in the absence of treatment. MRe is either the consequence of structural lesion in at least one component of the valve (i.e., primary MRe, often seen as a prolapse of the leaflet in the left atrium on the echocardiography) or a ventricular disease, such as myocardial infarction resulting in the loss of the leaflets’ contact (i.e., secondary MRe) [74]. Currently, the success of Mitral Valve reparation (MVr) is assessed using echocardiography, which provides a precise real-time morphological analysis and some functional parameters based on heuristic approximations. However, immediately after percutaneous-MVr procedures, up to 7% of the patients have moderate to severe regurgitation [75]. The incidence of MRe recurrence demonstrates the insufficient power of echocardiography to predict mid-term postoperative failure at the time of the repair. This substantial clinical limitation in the current standard of care represents the need for identifying new prognostic parameters and for developing unique tools to measure these new parameters. Such tools used in addition to echocardiography could allow physicians to significantly improve their abilities to perform effective and long-lasting repairs at a greater frequency. 

**Figure 14:**
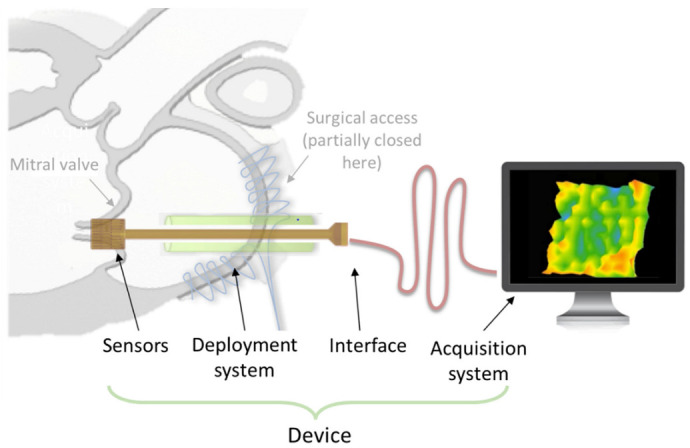
Working principle of the biosensor device used for force measurement of mitral valve coaptation.

In a recent study, our team have demonstrated the possibility of measuring the coaptation force in MV, defined as the contact between the two mitral leaflets during systole [76]. This contact is the ultimate “goal” of the whole mitral system, since it ensures its proper sealing function. MV restoration of a perfect coaptation has revealed to be highly correlated with better long MV regurgitation outcomes [77]. The “creation of a marge surface of coaptation” is one of the three fundamental principles of MV reconstruction formulated by Pr. Alain Carpentier decades ago [78]. Although it has been known for decades that obtaining a large coaptation surface promotes optimal clinical long-term outcomes, the actual phenomenon explaining this key role is not well understood. Recent scientific and technological innovation open up the opportunity to develop biosensors allowing a precise measurement of coaptation forces along the coaptation surface in order to obtain a mapping of these forces that could make real-time measurement of MV coaptation possible. The developed device must acquire instantaneous measurements and accurately display the valvular closure force. As described Figure 14, the system is composed of three main following parts:

- High-resolution sensors enable the measurement of pressure inside the MV, with typical amplitude of stress of around 0.1 MPa to 0.7 MPa [79]. The sensors must be no thicker than 200 µm with low stiffness to “mold” to the shape of the MV without interacting with its function.
- A deployment system allows the sensors to be set and manipulated inside of the valve during the measurement period but also to be retrieved outside of the heart after the measurement.
- Acquisition system allows for real-time recording, analyzing and monitoring of the data.

Our first prototype was built using piezoresistive elements with the aim of demonstrating the possibility of measuring the force of coaptation, which is considered to be a key parameter to help the understanding of MV pathology [76]. The piezoresistive materials have been revealed to be easily processed, cost effective, miniaturizable and adaptable to printing technology [80,81], which makes them one of the most widely used in sensing devices, principally as strain gage. However, contrarily to the piezoelectric active element, the strain gage is passive and thus needs a conditioning circuit to convert a resistance variation to an output voltage. The output signal is somehow prone to noise because of the poor signal-to-noise ratio. Accordingly, the signal induced from the strain gage is usually filtered to remove high-frequency noises, resulting in signal distortion due to harmonics alteration. The signal is commonly acquired via a quarter-bridge configuration where the output voltage (Δu) is proportional to the input strain (*S*):

(1) $$\Delta u=\frac{V_{CC}}{4}\times GF\times S$$

where *V_cc_* and *GF* denote the supplied voltage and the gage factor, respectively. The *GF* of the most commercially available strain sensors is quite small (i.e., equal to 2), leading to very low Δu signal compared to the output directly acquired from the piezoelectric sensor [82]. Better sensitivity of the strain gage can be enhanced by selecting a higher *GF*, but the sensor is less linear and less stable versus the temperature variation.

Other limitations of the piezoresistive materials are the drift effect (meaning derived signal in a long-time measure) and nonlinear response, especially when being subjected to a bending excitation driven by the MV [76]. Piezoelectric materials, however, were demonstrated to be efficient not only in compression force [82,83] but also in bending force [84,85]. Moreover, these materials deliver a voltage signal merely in case of mechanical solicitation, which in turn results in no drift effect. Such exceptional features undoubtedly make piezoelectric sensors promising candidates for the characterization of MV, which could overcome the technological lock of the first sensor networks’ prototype. The data of Figure 12a obtained in this work confirm a good correlation between mechanical stress and the piezoelectric response. Interestingly, the amplitude of the applied stress (i.e., around 0.1 MPa) corresponds to the typical value obtained from the modeling reported by Rim et al. [79]. As illustrated in Figure 12b–d, electromechanical coupling of the ZnO composites is quite linear, which is essential for the design of highly precise force mapping. Regarding the mechanical characteristics (cf., Figure 9), the developed material exhibits suitable Young modulus range (~10 MPa) that allows for a good mechanical adaptation between the sensor and the MV [86]. Finally, the piezoelectric and mechanical properties of the composites are considered key parameters in the design of force sensors. 

## 5. Conclusions

This work demonstrates the innovative combination of CBD with DEP processes to fabricate structured (i.e., aligned) ZnO MR/PDMS composites with a strong improvement of the mechanical, dielectric and piezoelectric properties. This work firstly focused on the microstructure of flexible composites, in particular the AR of ZnO fillers that can be related to the dielectric and piezoelectric properties of the composites. Indeed, a higher level of alignment was achieved within the curing matrix containing higher AR fillers, which were found to rotate with their long axes lying in the direction of the electric field before coalescing to form chains. Adequate parameters of the external electric field (0.6 V/μm, 2 Hz) were selected for the implementation of the DEP process, with the aim of optimizing the filler’s alignment. Random composites integrating 8% vol. MR-shaped fillers, thanks to their higher AR than the MP counterparts, led to a 2-fold increase in both dielectric and piezoelectric responses. This improvement is more obvious when the MR composite was structured via the DEP process, giving rise to, respectively, 1.3-fold, 5-fold and 2.6-fold enhancement in the mechanical, dielectric and piezoelectric properties, respectively, as opposed to the corresponding MP composite. The numerical model provided the electric field distribution maps of both MP and MR within the PDMS matrix, highlighting improved performance of the aligned MR-based composite. Morphological analysis using Raman scattering spectrum was revealed to be an effective approach in the identification of intrinsic/extrinsic point defects incorporated in ZnO MRs grown by CBD. The results confirmed that the appearance of high free electron density could engender a screen effect, which in turn might decrease the piezoelectric sensitivity of the composite. It was concluded that using thermal annealing throughout the chemical synthesis of ZnO MRs would be a solution to weaken the inevitable screen effect.

Besides being biocompatible, the developed material should also be sterilizable. In the near future, further considerations should be investigated to confirm whether or not the sterilization process would affect the material properties [87]. Parallelly, preliminary tests of the developed piezoelectric sensor in ex vivo simulator are envisaged to validate its reliability. The new insight of this research might be useful in assisting surgeons during MVr while providing real-time, objective measurements in support of the conventional intraoperative morphologic assessment [76]. Coaptation force characterization could also be valuable in improving the understanding of physical changes occurring during primary MRe development and after MVr, making it possible to predict long-term effects of postoperative outcomes. 

## Data Availability

Not applicable.
